# Effect of Acute Levodopa Up-Titration on Blood Pressure in Patients With Early Stage Parkinson’s Disease: Results of a Levodopa Challenge Test

**DOI:** 10.3389/fnagi.2021.778856

**Published:** 2022-01-03

**Authors:** Xiaoqin He, Chengjun Mo, Yi Zhang, Ying Cai, Xiaodong Yang, Yiwei Qian, Qin Xiao

**Affiliations:** Department of Neurology and Institute of Neurology, Ruijin Hospital, Shanghai Jiao Tong University School of Medicine, Shanghai, China

**Keywords:** Parkinson’s disease, levodopa, blood pressure, hypotension, levodopa challenge test

## Abstract

**Objective:** Levodopa up-titration is the primary therapeutic strategy as the Parkinson’s disease (PD) progresses. However, the effects of levodopa up-titration on blood pressure (BP) are inconclusive. This study aimed to investigate the effect of acute levodopa up-titration simulated by levodopa challenge test (LCT) on BP in patients with early stage PD.

**Methods:** We monitored BP in 52 patients with early stage PD using a standardized standing test. BP was assessed in supine position after 10 min of rest and at 1 and 3 min after standing up. BP was measured in the “off-state” and the best “on-state” during LCT in the morning at hospital. In another day, “off-state” and the best “on-state” BP was measured before and after anti-PD drug uptake in the morning at home. Demographic and clinical features of the patients were evaluated and analyzed.

**Results:** In the LCT, the prevalence of OH in the “off-state” and the best “on-state” was 11.5 and 13.5%, respectively. Additionally, the OH in the best “on-state” was associated with OH in the “off-state” and monoamine oxidase B inhibitor use. Although 38 (73.1%) patients experienced levodopa-induced hypotension during the LCT, no risk factors were identified. While BP reductions were observed after taking anti-PD drugs at home, no further reduction was seen during acute levodopa up-titration simulated by the LCT.

**Conclusion:** Our results demonstrate that acute levodopa up-titration does not exacerbate BP reduction induced by anti-PD drugs at home. BP monitoring is critical for the management of patients with PD.

## Introduction

Parkinson’s disease (PD) is the second most common neurodegenerative disease, affecting approximately 0.39% of the Chinese population above 50 years of age (Li et al., 2019). In addition to the classic motor symptoms (i.e., bradykinesia, rigidity, and resting tremor), attention has been increasingly paid to non-motor symptoms (NMS), including olfactory dysfunction, sleep disorders, anxiety, depression, and autonomic dysfunction in patients with PD (Postuma et al., 2015; Schapira et al., 2017). Autonomic dysfunction is a prominent symptom of PD, particularly cardiovascular autonomic dysfunction, which occurs in approximately 70% of patients with PD (Goldstein et al., 2000). The common cardiovascular autonomic dysfunctions associated with PD include blood pressure (BP) abnormalities, such as hypotension, particularly orthostatic hypotension (OH), which can occur at any phase of PD (Schapira et al., 2017). The estimated prevalence of OH in patients with PD is approximately 27.7%, ranging from 4.6 to 62.5% (Matsui et al., 2006; Baschieri et al., 2015; Mu et al., 2020). Hypotension manifests as dizziness, fatigue, blurred vision, and even falls in patients with PD, which could be a major cause of disability and a determinant of quality of life. Patients who lose self-care ability are a huge burden on caregivers and the health care economic system (Ricci et al., 2015; Fanciulli et al., 2020; Kim et al., 2020). Therefore, monitoring BP is a vital part of the daily management of patients with PD.

Hypotension is associated with a worse PD severity and postural instability/gait difficulty (PIGD) phenotype (Allcock et al., 2006; Quarracino et al., 2020). Autonomic dysfunction progresses with disease severity, which worsens fragile BP homeostasis (Quarracino et al., 2020). Although drugs provide the most effective treatment for PD, anti-PD drugs, such as levodopa, dopamine agonists, and monoamine oxidase B (MAO-B) inhibitors are also associated with hypotension (Kujawa et al., 2000; Stryjer et al., 2005; Noack et al., 2014). Levodopa is the mainstay of medical therapy for motor symptoms at all stages of PD (Connolly and Lang, 2014), especially the advanced stage. Higher doses and more frequent administration of levodopa are inevitable as the disease progresses, and can lead to hypotension (Hiorth et al., 2019; Armstrong and Okun, 2020). Thus, it is a paradox that the avoidance of side effects counters satisfactory therapeutic effects. Direct systolic BP dipping in the supine and standing positions has been reported in a levodopa challenge test (LCT) without confirming the causal relationship between high-dose levodopa and BP dip (Fabbri et al., 2017). Under the circumstance of inevitable dose up-titration, there have been concerns over the BP-lowering effect of anti-PD drugs. Additionally, the risk factors for hypotension aggravated by levodopa up-dosage have not been reported.

In this study, LCT was used to simulate the rapidly increasing dose of levodopa in patients with early stage PD. BP was monitored through a standardized standing test in the “off-state” and best “on-state” following uptake of anti-PD drugs at home and LCT at hospital to evaluate the effects of acute levodopa up-titration on hypotension induced by the regular use of anti-PD drugs.

## Materials and Methods

### Participants

In this study, we enrolled 52 patients with PD from the Movement Disorder Clinic at the Department of Neurology, Ruijin Hospital, Shanghai Jiao Tong University School of Medicine. PD was diagnosed according to the United Kingdom Parkinson’s Disease Society Brain Bank Clinical Diagnostic criteria (Daniel and Lees, 1993).

The exclusion criteria were as follows: (1) secondary parkinsonism; (2) other central nervous system diseases; (3) history of stroke; (4) history of cardiovascular diseases; (5) history of surgical or medical treatment for cancer within the last 3 years; and (6) investigator judgment that the candidate was not suitable for participation in the study.

All patients provided written informed consent to participate in this study. The study was approved by the Ethics Committee of Ruijin Hospital, Shanghai Jiao Tong University School of Medicine.

### Levodopa Challenge Test

LCT was performed at hospital in the morning after withdrawal of all anti-PD drugs (i.e., dopamine agonists for at least 36 h; levodopa, and other anti-PD drugs for at least 12 h) to achieve an appropriate washout. In addition, antihypertensive drugs were also withdrawn in the morning. The levodopa challenge dosage was calculated as 150% of the regular morning levodopa equivalent dose (LED) (Tomlinson et al., 2010; Saranza and Lang, 2021). Based on the calculated levodopa challenge dosage, the patients received calculated tablets of 200/50 mg levodopa/benserazide.

### Blood Pressure Measurement

We measured BP with a validated BP monitoring device (OMRON HEM-7051, OMRON Inc., Dalian, China) with a standard bladder (13 cm wide and 30 cm long). BP was assessed using the standardized standing test. Patients with PD were well educated about the detail of standardized standing test when visited to the clinical center. Firstly, supine BP was measured in the supine position after 10 min of rest in a comfortable and safe environment, while the standing BP was measured at 1 and 3 min after standing up without external assistance. Meanwhile, symptoms of hypoperfusion, such as dizziness, sleepiness, and blurred vision, within 3 min of upright posture were also assessed. BP measurements of 52 patients were performed both at home and hospital. BP was measured in the “off-state” and the best “on-state” during LCT in the morning at hospital. In another day, “off-state” and the best “on-state” BP was measured before and after anti-PD drug uptake in the morning at home. The “off-state” was defined as the period when all anti-PD drugs were withheld for at least 12 h, and the best “on-state” was defined as the peak of anti-PD drugs benefit in the morning at home or the peak of levodopa benefit in the LCT at hospital. OH was defined as a reduction of systolic BP by at least 20 mmHg or diastolic BP by at least 10 mmHg within 3 min of standing up from the supine position (Freeman et al., 2011). Anti-PD drug-induced hypotension in the morning at home was defined, if either one of following criteria was met: (1) a decrease of systolic BP by at least 20 mmHg/diastolic BP by 10 mmHg from “off-state” to the best “on-state” in the supine position; (2) a decrease of systolic BP by at least 20 mmHg/diastolic BP by 10 mmHg from “off-state” to the best “on-state” in the 1-min/3-min standing position. Levodopa-induced hypotension during the LCT at hospital was defined, if either one of following criteria was met: (1) a decrease of systolic BP by at least 20 mmHg/diastolic BP by 10 mmHg from “off-state” to the best “on-state” in the supine position; (2) a decrease of systolic BP by at least 20 mmHg/diastolic BP by 10 mmHg from “off-state” to the best “on-state” in the 1-min/3-min standing position.

### Clinical Evaluations

Demographic data, history of hypertension, use of antihypertensive drugs, age of onset, Hoehn and Yahr stage, disease duration, and use of anti-PD drugs were recorded. Levodopa equivalent daily dose (LEDD) was calculated according to methods described in a previous study (Tomlinson et al., 2010). Clinical features were assessed using the Movement Disorder Society Unified Parkinson’s Disease Rating Scale (MDS-UPDRS). The MDS-UPDRS part III was assessed in the “off-state” and the best “on-state” during LCT. Levodopa responsiveness (%) was calculated as follows:


$$Levodopa\;Responsiveness\;\left(\%\right)\;=\;\frac{off\;state\;MDSUPDRS\;III\;scores-best\;on\;state\;MDSUPDRS\;III\;scores\;}{off\;state\;MDSUPDRS\;III\;scores}\;\times 100\%\;\left(\text{Saranza and Lang},\text{ 2}0\text{21}\right)$$

Non-motor symptoms, including depression, anxiety, autonomic functions, rapid eye movement (REM) sleep behavior disorder, sleep problems, quality of life, and cognitive function, were assessed using the Non-Motor Symptoms Quest Scale (NMS-Quest), Hamilton Depression Scale (HAMD-17), Hamilton Anxiety Rating Scale (HAMA), the Scale for Outcomes in Parkinson’s disease-Autonomic (SCOPA-AUT), REM Sleep Behavior Disorder Screening Questionnaire (RBD-SQ), Parkinson’s Disease Sleep Scale-2 (PDSS-2), 39-item Parkinson’s Disease Questionnaire (PDQ-39), Mini-Mental State Examination (MMSE), and Montreal Cognitive Assessment-Beijing version (MoCA). Motor phenotypes were divided into PIGD-dominant and non-PIGD-dominant based on the MDS-UPDRS scores (Stebbins et al., 2013).

### Statistical Analysis

Data are expressed as mean ± standard deviation (SD) for continuous variables or frequency (percentage) for categorical values. Continuous variables were analyzed using the Mann–Whitney *U*-test or paired Wilcoxon test, and categorical values were evaluated using the chi-squared test or McNemar test among the subgroups. Variables with a significant difference based on univariable logistic regression analysis were entered into multivariable logistic regression analysis. Multivariable logistic regression analysis was performed to investigate the risk factors associated with OH, anti-PD drug-induced hypotension, and levodopa-induced hypotension. Statistical significance was defined as a *P*-value < 0.05. Statistical analyses were performed using Statistical Product and Service Solutions software (IBM SPSS Statistics 22.0; SPSS Inc., Chicago, IL, United States).

## Results

### Demographic and Clinical Characteristics of Patients With Parkinson’s Disease

**FIGURE 1:**
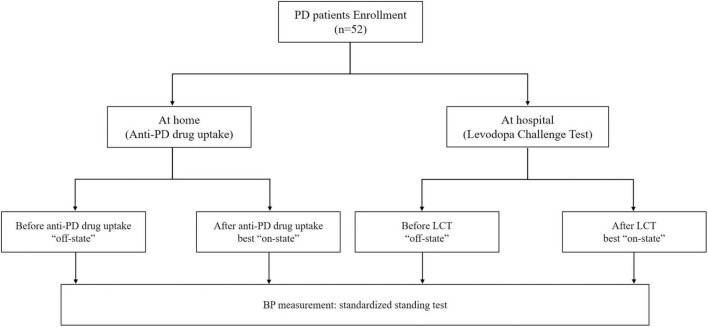
Flow diagram of the study. At home: “off-state,” defined as the period when all anti-PD drugs were withdrawn for at least 12 h; best “on-state,” defined as the peak of anti-PD drugs benefit in the morning at home; At hospital: “off-state,” defined as the period when all anti-PD drugs were withdrawn for at least 12 h; best “on-state,” defined as the peak of levodopa benefit in the morning at hospital; PD, Parkinson’s disease; LCT, levodopa challenge test; BP, blood pressure.

A total of 52 patients with early stage PD were enrolled in this study (Figure 1). The BP measurements of these patients were performed both at home and hospital. The demographics and clinical characteristics of these participants are shown in Table 1. Fifty patients received levodopa treatment, of whom 42 received only levodopa/benserazide, seven received levodopa/benserazide and controlled-release levodopa/carbidopa, and one patient received only controlled-release levodopa/carbidopa. Of the 37 patients who used dopamine agonists, 32 used pramipexole and five used piribedil. Among the six patients who received MAO-B inhibitors, five received selegiline, and one received rasagiline. Only three patients with PD received catechol-O-methyltransferase (COMT) inhibitor (entacapone).

**TABLE 1:** Demographic and clinical characteristics of patients with PD.

Characteristics	Total
Number	52
Gender (male/female)	30/22
Age (years)	65.19 ± 8.80
BMI (kg/m^2^)	23.76 ± 2.47
History of hypertension (n, %)	22 (42.3)
Antihypertensive drugs (n, %)	15 (28.8)
Age of onset (years)	60.25 ± 8.38
Disease duration (years)	4.93 ± 3.24
Hoehn and Yahr stage	2.28 ± 0.41
PIGD-dominant (n, %)	25 (48.1)
MDS-UPDRS I score	9.38 ± 4.57
MDS-UPDRS II score	12.29 ± 5.50
“Off-state” MDS-UPDRS III score	32.52 ± 11.13
“Off-state” MDS-UPDRS total score	54.50 ± 17.90
NMS-Quest score	7.92 ± 3.86
SCOPA-AUT cardiovascular domain score	0.38 ± 0.97
SCOPA-AUT gastrointestinal domain score	4.04 ± 3.33
SCOPA-AUT total score	9.02 ± 6.95
HAMD-17 score	5.67 ± 4.48
HAMA score	7.38 ± 4.79
RBD-SQ score	2.04 ± 2.90
PDSS-2 score	9.40 ± 6.12
PDQ-39 score	22.77 ± 15.73
MMSE score	27.48 ± 3.20
MoCA score	22.75 ± 5.14
LEDD (mg)	471.62 ± 208.73
**Anti-PD drugs**	
Levodopa (n, %)	50 (96.2)
Dopamine agonists (n, %)	37 (71.2)
MAO-B inhibitors (n, %)	6 (11.5)
COMT inhibitor (n, %)	3 (5.8)
Amantadine (n, %)	3 (5.8)
Benzhexol (n, %)	3 (5.8)

*Data were shown as mean ± SD or frequency (percentage). PD, Parkinson’s disease; BMI, Body Mass Index; PIGD, postural instability/gait difficulty; MDS-UPDRS, Movement Disorder Society-Unified Parkinson’s Disease Rating Scale, “off-state” MDS-UPDRS III and total score were evaluated in levodopa challenge test; “off-state,” defined as the period when all anti-PD drugs were withdrawn for at least 12 h; NMS-Quest, Non-motor Symptoms Quest Scale; SCOPA-AUT, Scale for Outcomes in Parkinson’s Disease-Autonomic; HAMD-17, Hamilton Depression Scale; HAMA, Hamilton Anxiety Rating Scale; RBD-SQ, Rapid Eye Movement (REM) Sleep Behavior Disorder Screening Questionnaire; PDSS-2, Parkinson’s Disease Sleep Scale-2; PDQ-39, 39-item Parkinson’s Disease Questionnaire; MMSE, Mini Mental State Examination; MoCA, Montreal Cognitive Assessment; LEDD, levodopa equivalent daily dosage; MAO-B, monoamine oxidase B; COMT, catechol-O-methyltransferase.*

### Prevalence and Clinical Features of Orthostatic Hypotension and Anti-Parkinson’s Disease Drug-Induced Hypotension at Home

Seven patients had OH in the “off-state” before taking anti-PD drugs in the morning at home (Supplementary Table 1). These patients had higher HAMD-17 (9.14 ± 5.08 vs. 5.13 ± 4.18, *P* = 0.043) and HAMA (11.43 ± 5.13 vs. 6.76 ± 4.47, *P* = 0.026) scores. In addition, there were more patients with OH in the “off-state” endured OH in the best “on-state” (57.1% vs. 6.7%, *P* = 0.004). Seven patients had OH in the best “on-state.” No difference was seen in the clinical features between patients with or without OH in the best “on-state.” Anti-PD drug-induced hypotension was observed in 31 patients in the best “on-state.” PIGD-dominant patients had more anti-PD drug-induced hypotension compared to the non-PIGD-dominant patients (64.5% vs. 23.8%, *P* = 0.004).

Univariable logistic regression analysis found that the HAMD-17 [odds ratio (OR) = 1.20, 95% confidence interval (CI): 1.01–1.42, *P* = 0.039] and HAMA (OR = 1.22, 95% CI: 1.02–1.45, *P* = 0.026) scores were associated with OH in the “off-state” at home, and these associations were not persisted in multivariable logistic regression analysis. Based on univariable and multivariable logistic regression analysis, only OH in the “off-state” independently correlated with OH in the best “on-state” at home (OR = 18.67, 95% CI: 2.79–124.90, *P* = 0.003). Only PIGD-dominant (OR = 5.82, 95% CI: 1.68–20.20, *P* = 0.006) was a risk factor for anti-PD drug-induced hypotension based on univariable and multivariable logistic regression analysis (Table 2).

**TABLE 2:** Logistic regression analysis of the risk factors related to OH and anti-PD drug-induced hypotension at home.

Characteristics	“Off-state” OH				Best “on-state” OH				Anti-PD drug-induced hypotension			
	Univariable OR	*P*-value	Multivariable OR	*P*-value	Univariable OR	*P*-value	Multivariable OR	*P*-value	Univariable OR	*P*-value	Multivariable OR	*P*-value
	(95% CI)		(95% CI)		(95% CI)		(95% CI)		(95% CI)		(95% CI)	
PIGD-dominant	–	–	–	–	–	–	–	–	5.82 (1.68, 20.20)	**0.006**	5.82 (1.68, 20.20)	**0.006**
HAMD-17	1.20 (1.01, 1.42)	**0.039**	1.01 (0.69, 1.47)	0.981	–	–	–	–	–	–	–	–
HAMA	1.22 (1.02, 1.45)	**0.026**	1.21 (0.83, 1.77)	0.319	–	–	–	–	–	–	–	–
Home “off-state” OH	–	–	–	–	18.67 (2.79, 124.90)	**0.003**	18.67 (2.79, 124.90)	**0.003**	–	–	–	–

*Demographic and clinical features were assessed as risk factors using logistic regression analysis. Each feature was analyzed by univariable logistic regression analysis. Only significant variables (P < 0.05) based on univariable logistic regression analysis were analyzed using multivariable logistic regression analysis. P-values with statistic significance (<0.05) are in bold. OH, orthostatic hypotension; PD, Parkinson’s disease; “off-state,” defined as the period when all anti-PD drugs were withdrawn for at least 12 h; best “on-state,” defined as the peak of anti-PD drugs benefit in the morning at home; anti-PD drug-induced hypotension in the morning at home was defined, if either one of following criteria was met: (1) a decrease of systolic BP by at least 20 mmHg/diastolic BP by 10 mmHg from “off-state” to the best “on-state” in the supine position; (2) a decrease of systolic BP by at least 20 mmHg/diastolic BP by 10 mmHg from “off-state” to the best “on-state” in the 1-min/3-min standing position; OR, odds ratio; CI, confidence interval; PIGD, postural instability/gait difficulty; HAMD-17, Hamilton Depression Scale; HAMA, Hamilton Anxiety Rating Scale.*

### Prevalence and Clinical Features of Orthostatic Hypotension and Levodopa-Induced Hypotension in the Levodopa Challenge Test

LCT was performed in 52 patients with PD with a mean challenge dosage of 241.39 ± 88.38 mg. The MDS-UPDRS III score improved by 14.71 ± 5.56 points, going from 32.52 ± 11.13 in the “off-state” to 17.81 ± 7.76 in the best “on-state” (*P* < 0.001). Based on the MDS-UPDRS III scores, the average levodopa responsiveness was 45.98 ± 11.41%. Six patients had OH in the “off-state” before LCT and higher MDS-UPDRS III (40.17 ± 5.64 vs. 31.52 ± 11.32, *P* = 0.018) and total (65.83 ± 11.89 vs. 53.02 ± 18.11, *P* = 0.035) scores only in the “off-state” (Supplementary Table 2). They also had worse autonomic symptoms in accordance with their higher SCOPA−AUT gastrointestinal domain (7.00 ± 4.34 vs. 3.65 ± 3.03, *P* = 0.048) and total (15.67 ± 8.48 vs. 8.15 ± 6.33, *P* = 0.028) scores. A higher HAMA score was also observed in these patients (11.83 ± 4.31 vs. 6.80 ± 4.57, *P* = 0.017). Patients with OH in the “off-state” during the LCT also had a higher prevalence of OH in the “off-state” (66.7% vs. 6.5%, *P* = 0.002) and the best “on-state” (50.0% vs. 8.7%, *P* = 0.026) at home. Symptoms of hypoperfusion were more frequent in patients with OH than in those without OH in the “off-state” (66.7% vs. 4.3%, *P* = 0.001). Patients with OH in the “off-state” also had a higher incidence of OH (50.0% vs. 8.7%, *P* = 0.026) and symptoms of hypoperfusion (66.7% vs. 13.0%, *P* = 0.010) in the best “on-state.”

The prevalence of OH in the best “on-state” during LCT was 13.5%. More patients with OH in the best “on-state” used MAO-B inhibitors (42.9% vs. 6.7%, *P* = 0.026) and also had OH in the “off-state” during LCT (42.9% vs. 6.7%, *P* = 0.026). No differences in other clinical characteristics were observed in patients with and without OH in the best “on-state” during LCT.

Of the 52 patients with PD who underwent LCT, 38 had levodopa-induced hypotension. These 38 patients were older (66.82 ± 8.25 vs. 60.79 ± 9.02, *P* = 0.019), had a longer disease duration (5.62 ± 3.32 vs. 3.07 ± 2.16, *P* = 0.007), higher MDS-UPDRS III scores (34.63 ± 10.82 vs. 26.79 ± 10.21, *P* = 0.022), and higher total scores in the “off-state” (57.42 ± 18.18 vs. 46.57 ± 14.96, *P* = 0.049). Patients with levodopa-induced hypotension during LCT had a higher LEDD (515.44 ± 188.75 vs. 352.68 ± 220.64, *P* = 0.013).

The univariable logistic regression analysis found that OH in the “off-state” during LCT was significantly associated with higher SCOPA−AUT gastrointestinal domain (OR = 1.42, 95% CI: 1.02–1.97, *P* = 0.037), total (OR = 1.17, 95% CI: 1.02–1.34, *P* = 0.025), and HAMA (OR = 1.23, 95% CI: 1.03–1.49, *P* = 0.027) scores (Table 3). However, these associations did not emerge to be significant in the multivariable regression analysis. MAO-B inhibitors use and OH in the “off-state” of LCT were significantly related to OH in the best “on-state” of LCT based on univariable (both OR = 10.50, 95% CI: 1.57–70.25, *P* = 0.015) and multivariable (both OR = 14.64, 95% CI: 1.65–130.25, *P* = 0.016) regression analyses. Levodopa-induced hypotension in LCT was significantly associated with older age (OR = 1.09, 95% CI: 1.01–1.18, *P* = 0.034), longer disease duration (OR = 1.44, 95% CI: 1.06–1.96, *P* = 0.019), higher MDS-UPDRS III scores (OR = 1.09, 95% CI: 1.01–1.17, *P* = 0.030), higher NMS-Quest (OR = 1.25, 95% CI: 1.01–1.55, *P* = 0.044) and higher LEDD (OR = 1.00, 95% CI: 1.00–1.01, *P* = 0.018). However, the multivariable analysis found no significant association between these clinical features and levodopa-induced hypotension.

**TABLE 3:** Logistic regression analysis of the risk factors related to OH and levodopa-induced hypotension in levodopa challenge test.

Characteristics	“Off-state” OH				Best “on-state” OH				Levodopa-induced hypotension			
	Univariable OR	*P*-value	Multivariable OR	*P*-value	Univariable OR	*P*-value	Multivariable OR	*P*-value	Univariable OR	*P*-value	Multivariable OR	*P*-value
	(95% CI)		(95% CI)		(95% CI)		(95% CI)		(95% CI)		(95% CI)	
Age	–	–	–	–	–	–	–	–	1.09 (1.01,1.18)	**0.034**	1.05 (0.96,1.15)	0.284
Disease duration	–	–	–	–	–	–	–	–	1.44 (1.06,1.96)	**0.019**	1.13 (0.76,1.68)	0.541
“Off-state” MDS-UPDRS III part	–	–	–	–	–	–	–	–	1.09 (1.01,1.17)	**0.030**	1.02 (0.93,1.12)	0.741
NMS-Quest	–	–	–	–	–	–	–	–	1.25 (1.01,1.55)	**0.044**	1.14 (0.90,1.45)	0.283
SCOPA-AUT gastrointestinal domain	1.42 (1.02, 1.97)	**0.037**	1.29 (0.76,2.20)	0.350	–	–	–	–	–	–	–	–
SCOPA-AUT total	1.17 (1.02,1.34)	**0.025**	1.02 (0.80, 1.30)	0.879	–	–	–	–	–	–	–	–
HAMA	1.23 (1.03, 1.49)	**0.027**	1.18 (0.93, 1.49)	0.166	–	–	–	–	–	–	–	–
LEDD	–	–	–	–	–	–	–	–	1.00 (1.00, 1.01)	**0.018**	1.00 (1.00,1.01)	0.300
MAO-B inhibitors	–	–	–	–	10.50 (1.57, 70.25)	**0.015**	14.64 (1.65, 130.25)	**0.016**	–	–	–	–
LCT “off-state” OH	–	–	–	–	10.50 (1.57, 70.25)	**0.015**	14.64 (1.65, 130.25)	**0.016**	–	–	–	–

*Demographic and clinical features were assessed as risk factors using logistic regression analysis. Each feature was analyzed by univariable logistic regression analysis. Only significant clinical features (P < 0.05) based on univariable logistic regression analysis were analyzed using multivariable logistic regression analysis. P-values with statistic significance (<0.05) are in bold. OH, orthostatic hypotension; “off-state,” defined as the period when all anti-PD drugs were withdrawn for at least 12 h; best “on-state,” defined as the peak of levodopa benefit in the levodopa challenge test at hospital; levodopa-induced hypotension in the morning at hospital was defined, if either one of following criteria was met: (1) a decrease of systolic BP by at least 20 mmHg/diastolic BP by 10 mmHg from “off-state” to the best “on-state” in the supine position; (2) a decrease of systolic BP by at least 20 mmHg/diastolic BP by 10 mmHg from “off-state” to the best “on-state” in the 1-min/3-min standing position; OR, odds ratio; CI, confidence interval; MDS-UPDRS, Movement Disorder Society-Unified Parkinson’s Disease Rating Scale, “off-state” MDS-UPDRS III and total score were evaluated in levodopa challenge test; NMS-Quest, Non-motor Symptoms Quest Scale; SCOPA-AUT, Scale for Outcomes in Parkinson’s Disease-Autonomic; HAMD-17, Hamilton Depression Scale; HAMA, Hamilton Anxiety Rating Scale; LEDD, levodopa equivalent daily dosage; MAO-B, monoamine oxidase B.*

### Comparison of Blood Pressure Variation Between Anti-Parkinson’s Disease Drugs Uptake at Home and Levodopa Challenge Test

The influence of anti-PD drug uptake in the morning at home vs. LCT at hospital on BP was examined in this study (Table 4). Anti-PD drugs taken at home in the morning significantly reduced the systolic/diastolic BP in both the supine and standing postures (*P* < 0.001). And the BP change after orthostatism was not exacerbated after taking anti-PD drugs in the morning at home. In addition, no difference was found in the frequency of OH between “off-state” and the best “on-state” (13.5% vs. 13.5%, *P* = 1.000).

**TABLE 4:** BP variation during anti-PD drug uptake at home and levodopa challenge test.

BP (mmHg)	Anti-PD drug uptake in the morning at home						Levodopa challenge test at hospital							BP change from “off-state” to best “on-state” in home vs. hospital
		
	“Off-state”		Best “on-state”		BP value in “off- state” vs. best “on-state”	BP change by posture in “off- state” vs. best “on-state”		“Off-state”		Best “on-state”		BP value in “off- state” vs. best “on-state”	BP change by posture in “off- state” vs. best “on-state”	
					
	BP Value	BP change by posture	BP Value	BP change by posture			BP Value		BP change by posture	BP Value	BP change by posture			
**Supine position**														
SBP	138.60 ± 20.15	–	124.08 ± 18.25	–	<**0.001**	–	132.35 ± 18.50		–	117.40 ± 16.35	–	<**0.001**	–	0.518
DBP	81.50 ± 8.66	–	73.06 ± 9.42	–	<**0.001**	–	78.69 ± 8.96		–	69.33 ± 8.75	–	<**0.001**	–	0.588
**Standing 1-min**														
SBP	140.62 ± 21.72	2.02 ± 17.06	123.46 ± 21.55	−0.62 ± 16.19	<**0.001**	0.241	132.37 ± 20.19		0.02 ± 15.19	112.98 ± 18.58	−4.42 ± 14.31	<**0.001**	**0.021**	0.384
DBP	85.73 ± 10.51	4.23 ± 9.16	76.87 ± 12.62	3.81 ± 9.15	<**0.001**	0.475	83.37 ± 9.89		4.67 ± 7.67	72.21 ± 10.01	2.88 ± 8.20	<**0.001**	**0.029**	0.130
**Standing 3-min**														
SBP	140.48 ± 22.63	1.88 ± 16.63	124.67 ± 21.35	0.60 ± 14.40	<**0.001**	0.569	130.35 ± 17.54		−2.00 ± 14.02	114.77 ± 18.30	−2.63 ± 14.69	<**0.001**	0.530	0.942
DBP	86.54 ± 10.38	5.04 ± 8.8	77.81 ± 12.20	4.75 ± 8.49	<**0.001**	0.904	83.17 ± 9.10		4.48 ± 7.24	72.23 ± 9.10	2.90 ± 7.57	<**0.001**	0.111	0.137

*Data were shown as mean ± SD. Difference between subgroups were performed using paired comparison by Wilcoxon test. BP change by posture was defined as BP change after orthostatism in the standardized standing test; BP value in “off-state” vs. best “on-state” was defined as the comparisons of absolute BP in corresponding position in the “off-state” and that in the best “on-state” (both at home and hospital); BP difference by posture in “off- state” vs. best “on-state” was defined as comparisons of BP change after orthostatism between “off-state” and best “on-state” (both at home and hospital); Difference of BP change from “off-state” to best “on-state” in home vs. hospital was defined as the comparisons of absolute BP change from “off-state” to best “on-state” between home and hospital; P-values with statistic significance (<0.05) are in bold. PD, Parkinson’s disease; BP, blood pressure; SBP, systolic blood pressure; DBP, diastolic blood pressure; “off-state,” defined as the period when all anti-PD drugs were withdrawn for at least 12 h; best “on-state,” defined as the peak of anti-PD drugs benefit in the morning at home or the peak of levodopa benefit in the levodopa challenge test at hospital.*

Similarly, taking the levodopa challenge dosage in the morning at hospital also significantly reduced the systolic/diastolic BP in the supine and standing positions (*P* < 0.001). In the best “on-state,” the amplitude of systolic (*P* = 0.021) and diastolic (*P* = 0.029) BP fall after orthostatism in the 1-min standing position was larger than those in the “off-state.” However, the frequency of OH in the best “on-state” did not increase compared with that in the “off-state” (13.5% vs. 11.5%, *P* = 1.000). A 1.5-fold regular morning LED for the LCT caused no significant change in the BP reduction in the supine or standing position compared with anti-PD drug uptake in the morning at home. Additionally, there was no difference in the frequency between levodopa-induced hypotension at hospital and anti-PD drug induced-hypotension at home (73.1% vs. 59.6%, *P* = 0.167).

## Discussion

Regular anti-PD drugs taken at home caused a drop in BP. Acute levodopa up-titration during the LCT did not further exacerbate the anti-PD drug-induced hypotension. These results provide evidence for the safety of levodopa applications.

An increase in levodopa dosage and the frequency of its administration are inevitable with the progression of PD. However, aggravation of hypotension in response to increased levodopa dosage has not been confirmed. Fabbri et al. (2017) have reported a decrease in systolic BP (by 23 mm Hg) in the supine and (by 26 mm Hg) at 3-min standing positions from the “off-state” to the best “on-state” during an LCT. In another study, the supine systolic BP was reduced by approximately 19 mmHg after 200/50 mg levodopa/benserazide uptake (Noack et al., 2014). However, neither study reported BP data at home, which was affected by the regular use of anti-PD drugs. Thus, the additional effect of incremental levodopa dosage compared to the regular use of anti-PD drugs on BP has not yet been answered. In this study, we investigated the BP variation in patients with early stage PD following anti-PD drug uptake at home and acute levodopa up-titration simulated by LCT at hospital. Acute levodopa up-titration during the LCT caused both the systolic and diastolic BP to drop, and the amplitude of the drop was comparable to that caused by the regular use of anti-PD drugs at home. And no difference in the frequency between levodopa-induced hypotension and anti-PD drug-induced hypotension. These results demonstrate that acute levodopa up-titration does not aggravate the drop in BP. In conclusion, acute levodopa up-titration in patients with early stage PD did not aggravate the hypotension induced by the regular use of anti-PD drugs. In addition, we show an association between the PIGD phenotype and anti-PD drug-induced hypotension at home, which has not been reported in previous studies. We also found that the prevalence of drug-induced hypotension was higher than OH. Thus, we recommend paying attention to drug-induced hypotension, especially in patients with the PIGD phenotype. Patients with PD should be well educated about the importance of home BP monitoring and the detail BP assessments of PD, including the standardized standing test, when visited to the clinical center. And the BP monitoring is recommended before and 1–2 h after taking regular anti-PD drugs. Specially, BP record should be added into the diary of patients with PD. New portable BP monitoring devices, such as wrist-cuff BP devices, are warranted for the convenience and compliance of the patients.

In addition to drug-induced hypotension, we also evaluated the prevalence and risk factors for OH in patients with PD. The incidence of both OH in the “off-state” and the best “on-state” with regular anti-PD drug uptake at home was 13.5%, while that during the LCT was 11.5 and 13.5%, respectively. Our results were consistent with those of a previous study that reported an incidence of 15.9% in patients with PD using the same OH criteria (Nataraj and Rajput, 2005). However, another study found that up to 62.5% of patients with PD had OH, which is remarkably higher than our results (Matsui et al., 2006). Compared to our cohort, the patients in their study (Matsui et al., 2006) had a longer disease duration (4.93 ± 3.24 vs. 9.7 ± 6.3 years) and higher Hoehn and Yahr stage (2.28 ± 0.41 vs. 3.3 ± 0.4), suggesting more severe PD, which may have accounted for the higher OH incidence. While many studies have investigated the prevalence and risk factors of OH in PD, the results varied widely. Although the reported prevalence of OH in patients with PD ranged from 4.6 to 62.5% (Matsui et al., 2006; Baschieri et al., 2015), a meta-analysis of 19 studies reported that it was approximately 27.7% (Mu et al., 2020). This heterogeneity in the results could be due to the following reasons: (1) different OH diagnostic criteria, including the threshold for BP reduction with position change and different standing times; (2) patients with PD with variable disease conditions; and (3) diverse sample sizes. This study found that OH in the “off-state” was a risk factor for OH in the best “on-state.” In addition, MAO-B inhibitor use was also a risk factor for OH in the best “on-state” during the LCT. The previously reported risk factors for OH included male sex, older age, PIGD phenotype, poor gastrointestinal dysfunction, polypharmacy (defined as intake of > 5 medications), and the use of anti-PD drugs (e.g., levodopa, dopamine agonists, and MAO-B inhibitors) (Churchyard et al., 1999; Kujawa et al., 2000; Allcock et al., 2006; Perez-Lloret et al., 2012; Noack et al., 2014; Szewczyk-Krolikowski et al., 2014; Hiorth et al., 2019; Quarracino et al., 2020). Except MAO-B inhibitors, the other risk factors were not identified in our study. Previous studies reported changes in plasma levels of noradrenaline and adrenaline in patients with PD who used MAO-B inhibitors (Stryjer et al., 2005; Pursiainen et al., 2007), which regulate BP (Ricci et al., 2015; Umehara et al., 2018). These findings may explain the association between OH and the use of MAO-B inhibitors. Though the frequency of OH did not increase following the levodopa challenge dosage, BP fall after orthostatism was worse in the 1-min upright position in the best “on-state” than that in the “off-state” during LCT. Therefore, patients should be rigorously educated to stand up slowly to avoid falls and syncope caused by swift position change when up-titrating the levodopa dosage. It is also worth mentioning that about 11–16% of patients with PD who did not meet the OH diagnostic criteria presented with symptoms of hypoperfusion, highlighting the importance of BP monitoring for patients with PD, especially those suffering from hypoperfusion symptoms.

This study has several limitations. First is the small sample size. Second, this was a cross-sectional study, and prospective studies are warranted to validate our findings. Third, the recruited patients mainly had early stage PD. Further studies including patients with both early- and advanced-stage PD are needed.

In conclusion, our study demonstrated no exacerbation of hypotension in early stage patients regularly using anti-PD drugs following an acute up-titration of levodopa, suggesting its safety. We also highlight the importance of BP monitoring in the daily management of patients with PD, particularly before and 1–2 h after taking regular anti-PD drugs.

## Data Availability Statement

The raw data supporting the conclusions of this article will be made available by the authors, without undue reservation.

## Ethics Statement

The studies involving human participants were reviewed and approved by the Ethics Committee of Ruijin Hospital, Shanghai Jiao Tong University School of Medicine. The patients/participants provided their written informed consent to participate in this study.

## Author Contributions

XH and CM: clinical analyses and manuscript writing. YZ and YC: data collection. XY: manuscript revision and financial support. YQ and QX: study design, project management, financial support, and manuscript revision. All co-authors contributed to revising the manuscript for intellectual content and approved the final version for publication.

## Conflict of Interest

The authors declare that the research was conducted in the absence of any commercial or financial relationships that could be construed as a potential conflict of interest.

## Publisher’s Note

All claims expressed in this article are solely those of the authors and do not necessarily represent those of their affiliated organizations, or those of the publisher, the editors and the reviewers. Any product that may be evaluated in this article, or claim that may be made by its manufacturer, is not guaranteed or endorsed by the publisher.
