# Supplementary material for: Effect of Acute Levodopa Up-Titration on Blood Pressure in Patients With Early Stage Parkinson’s Disease: Results of a Levodopa Challenge Test
Source: Front Aging Neurosci. 2022 Jan 3;13:778856. doi: 10.3389/fnagi.2021.778856 (PMC8761988; doi:10.3389/fnagi.2021.778856)
Supplement: Supplementary file 1 [file Data_Sheet_1.PDF]

Effect of Acute Levodopa Up-titration on Blood Pressure in Patients with Early-stage Parkinson's Disease: Results of a Levodopa Challenge Test

Supplementary table 1. Comparison of demographic and clinical features between PD subgroups during anti-PD drugs uptake at home

Characteristics	"off-state" OH (+)	"off-state" OH (-)	best "on-state" OH (+)	best "on-state" OH (-)	anti-PD drug-induced hypotension (+)	anti-PD drug-induced hypotension (-)
Number (%)	7 (13.5)	45 (86.5)	7 (13.5)	45 (86.5)	31(59.6)	21 (40.4)
Gender (male/female)	5/2	25/20	5/2	25/20	17/14	13/8
Age (years)	68.29 ± 6.63	64.71 ± 9.05	69.57 ± 4.35	64.51 ± 9.14	66.52 ± 8.98	63.24 ± 8.34
BMI (kg/m^2)	23.23 ± 1.64	23.84 ± 2.58	24.17 ± 1.67	23.70 ± 2.58	23.71 ± 2.41	23.84 ± 2.60
History of hypertension (n, %)	3 (42.9)	19 (42.2)	3 (42.9)	19 (42.2)	15 (48.4)	7 (33.3)
Antihypertensive drugs (n, %)	2 (28.6)	13 (28.9)	2 (28.6)	13 (28.9)	9 (29.0)	6 (28.6)
Age of onset (years)	65.14 ± 6.59	59.49 ± 8.43	64.43 ± 4.72	59.60 ± 8.66	61.10 ± 8.55	59.00 ± 8.15
Disease duration (years)	3.14 ± 2.12	5.21 ± 3.31	5.14 ± 2.61	4.90 ± 3.35	5.40 ± 3.60	4.24 ± 2.55
Hoehn & Yahr stage	2.50 ± 0.29	2.24 ± 0.42	2.50 ± 0.29	2.24 ± 0.42	2.34 ± 0.44	2.19 ± 0.37
PIGD-dominant (n, %)	4 (57.1)	21 (46.7)	5 (71.4)	20 (44.4)	20 (64.5)	5 (23.8)[##]
MDS-UPDRS I score	11.43 ± 3.64	9.07 ± 4.65	10.86 ± 3.89	9.16 ± 4.67	9.94 ± 4.21	8.57 ± 5.06
MDS-UPDRS II score	11.86 ± 3.93	12.36 ± 5.74	11.43 ± 4.28	12.42 ± 5.70	13.32 ± 5.95	10.76 ± 4.48
"off-state" MDS-UPDRS III score	33.86 ± 7.69	32.31 ± 11.63	35.43 ± 5.22	32.07 ± 11.76	34.19 ± 11.82	30.05 ± 9.77
best "on-state" MDS-UPDRS III score	20.57 ± 6.55	17.38 ± 7.91	21.00 ± 4.90	17.31 ± 8.04	18.77 ± 8.34	16.38 ± 6.75
"off-state" MDS-UPDRS total score	57.14 ± 12.35	54.09 ± 18.69	57.71 ± 9.86	54.00 ± 18.87	57.97 ± 19.39	49.38 ± 14.38
best "on-state" MDS-UPDRS total score	43.86 ± 11.08	39.16 ± 15.21	43.29 ± 9.25	39.24 ± 15.40	42.55 ± 15.90	35.71 ± 12.00
Levodopa responsiveness (%)	40.46 ± 9.30	46.83 ± 11.55	41.31 ± 8.70	46.70 ± 11.69	46.37 ± 11.75	45.40 ± 11.15
NMS-Quest score	8.00 ± 3.87	7.91 ± 3.91	8.29 ± 3.82	7.87 ± 3.91	8.52 ± 3.38	7.05 ± 4.42
SCOPA-AUT cardiovascular domain score	1.00 ± 1.29	0.29 ± 0.89	0.71 ± 1.25	0.33 ± 0.93	0.55 ± 1.18	0.14 ± 0.48
SCOPA-AUT gastrointestinal domain score	5.00 ± 3.87	3.89 ± 3.26	4.43 ± 3.55	3.98 ± 3.33	4.71 ± 3.41	3.05 ± 3.02
SCOPA-AUT total score	10.00 ± 7.33	8.87 ± 6.97	9.29 ± 6.37	8.98 ± 7.10	10.16 ± 6.64	7.33 ± 7.22

HAMD-17 score	9.14 ± 5.08	5.13 ± 4.18*	7.29 ± 5.91	5.42 ± 4.24	5.23 ± 4.11	6.33 ± 4.99
HAMA score	11.43 ± 5.13	6.76 ± 4.47*	9.43 ± 6.45	7.07 ± 4.48	7.45 ± 4.06	7.29 ± 5.80
RBD-SQ score	2.57 ± 3.36	1.96 ± 2.85	2.71 ± 3.45	1.93 ± 2.83	2.00 ± 2.86	2.10 ± 3.02
PDSS-2 score	10.43 ± 4.65	9.24 ± 6.34	9.86 ± 4.91	9.33 ± 6.33	9.19 ± 5.46	9.71 ± 7.11
PDQ-39 score	21.43 ± 17.15	22.98 ± 15.70	16.57 ± 14.23	23.73 ± 15.88	24.61 ± 15.65	20.05 ± 15.82
MMSE score	26.43 ± 4.35	27.64 ± 3.01	26.71 ± 4.46	27.60 ± 3.00	27.48 ± 2.94	27.48 ± 3.61
MoCA score	21.43 ± 5.59	22.96 ± 5.11	23.00 ± 5.89	22.71 ± 5.09	22.77 ± 4.64	22.71 ± 5.93
LEDD (mg)	467.86 ± 180.11	472.20 ± 214.65	426.79 ± 204.94	478.59 ± 210.72	513.32 ± 188.50	410.06 ± 226.12
Anti-PD drugs						
Levodopa (n, %)	7 (100.0)	43 (95.6)	7 (100.0)	43 (95.6)	31 (100.0)	19 (90.5)
Dopamine agonists (n, %)	5 (71.4)	32 (71.1)	4 (57.1)	33 (73.3)	24 (77.4)	13 (61.9)
MAO-B inhibitors (n, %)	1 (14.3)	5 (11.1)	1 (14.3)	5 (11.1)	2 (6.5)	4 (19.0)
COMT inhibitor (n, %)	0	3 (6.7)	0	3 (6.7)	2 (6.5)	1 (4.8)
Amantadine (n, %)	0	3 (6.7)	0	3 (6.7)	2 (6.5)	1 (4.8)
Benzhexol (n, %)	0	3 (6.7)	0	3 (6.7)	2 (6.5)	1 (4.8)
Home BP measurement						
“off-state” OH (n, %)	-	-	4 (57.1)	3 (6.7)$^{\$\$}$	6 (19.4)	1 (4.8)
“off-state” symptoms (n, %)	1 (14.3)	1 (2.2)	1 (14.3)	1 (2.2)	1 (3.2)	1 (4.8)
best “on-state” OH (n, %)	4 (57.1)	3 (6.7)**	-	-	6 (19.4)	1 (4.8)
best “on-state” symptoms (n, %)	2 (28.6)	6 (13.3)	3 (42.9)	5 (11.1)	6 (19.4)	2 (9.5)
anti-PD drug-induced hypotension (n, %)	6 (85.7)	25 (55.6)	6 (85.7)	25 (55.6)	-	-

Data were shown as mean ± SD or frequency (percentage). Differences between groups were assessed using the Mann–Whitney U test for numerical data and chi-squared test for categorical data.

* Difference in demographic and clinical features between patients with or without OH in the “off-state”, * P<0.05, ** P<0.01;

$^{\$}$ Difference in demographic and clinical features between patients with or without OH in the best “on-state”, $^{\$\$}$ P<0.01;

$^{\#}$ Difference in demographic and clinical features between patients with or without anti-PD drug-induced hypotension in the best “on-state”, $^{\#\#}$ P<0.01.

PD, Parkinson's disease; "off-state", defined as the period when all anti-PD drugs were withdrawn for at least 12 hours; OH, orthostatic hypotension; best "on-state", defined as the peak of anti-PD drugs benefit; anti-PD drug-induced hypotension, was defined, if either one of following criteria was met: (1) a decrease of systolic BP by at least 20 mmHg/diastolic BP by 10 mmHg from "off-state" to the best "on-state" in the supine position; (2) a decrease of systolic BP by at least 20 mmHg/diastolic BP by 10 mmHg from "off-state" to the best "on-state" in the 1-min/3-min standing position; BMI, Body Mass Index; PIGD, postural instability/gait difficulty; MDS-UPDRS, Movement Disorder Society-Unified Parkinson's Disease Rating Scale, "off-state" and best "on-state" MDS-UPDRS III and total score were evaluated in levodopa challenge test (LCT); levodopa responsiveness (%), defined as $Levodopa\ Responsiveness\ (\%) = \frac{off\ state\ MDS\ UPDRS\ III\ scores - best\ on\ state\ MDS\ UPDRS\ III\ scores}{off\ state\ MDS\ UPDRS\ III\ scores} \times 100\%$; NMS-Quest, Non-Motor Symptoms Quest Scale; SCOPA-AUT, Scale for Outcomes in Parkinson's Disease-Autonomic; HAMD-17, Hamilton Depression Scale; HAMA, Hamilton Anxiety Rating Scale; RBD-SQ, Rapid Eye Movement (REM) Sleep Behavior Disorder Screening Questionnaire; PDSS-2, Parkinson's Disease Sleep Scale-2; PDQ-39, 39-item Parkinson's Disease Questionnaire; MMSE, Mini Mental State Examination; MoCA, Montreal Cognitive Assessment; LEDD, levodopa equivalent daily dosage; MAO-B, monoamine oxidase B; COMT, catechol-O-methyltransferase; BP, blood pressure; symptoms, defined as dizziness, sleepiness, or blurred vision experienced by PD patients within 3 minutes in upright posture.
