# Supplementary material for: Effect of Acute Levodopa Up-Titration on Blood Pressure in Patients With Early Stage Parkinson’s Disease: Results of a Levodopa Challenge Test
Source: Front Aging Neurosci. 2022 Jan 3;13:778856. doi: 10.3389/fnagi.2021.778856 (PMC8761988; doi:10.3389/fnagi.2021.778856)
Supplement: Supplementary file 2 [file Data_Sheet_2.PDF]

# Effect of Acute Levodopa Up-titration on Blood Pressure in Patients with Early-stage Parkinson's Disease: Results of a Levodopa Challenge Test

Supplementary table 2. Comparison of demographic and clinical features between PD subgroups during levodopa challenge test

| Characteristics | "off-state" OH (+) | "off-state" OH (-) | best "on-state" OH (+) | best "on-state" OH (-) | levodopa-induced hypotension (+) | levodopa-induced hypotension (-) |
|---|---|---|---|---|---|---|
| Number (%) | 6 (11.5) | 46 (88.5) | 7 (13.5) | 45 (86.5) | 38 (73.1) | 14 (26.9) |
| Gender (male/female) | 4/2 | 26/20 | 5/2 | 25/20 | 20/18 | 10/4 |
| Age (years) | 71.33 ± 5.57 | 64.39 ± 8.86 | 69.71 ± 6.55 | 64.49 ± 8.95 | 66.82 ± 8.25 | 60.79 ± 9.02[#] |
| BMI (kg/m$^2$) | 22.52 ± 1.68 | 23.92 ± 2.52 | 22.94 ± 2.12 | 23.89 ± 2.52 | 23.66 ± 2.63 | 24.03 ± 2.03 |
| History of hypertension (n, %) | 2 (33.3) | 20 (43.5) | 3 (42.9) | 19 (42.2) | 17 (44.7) | 5 (35.7) |
| Antihypertensive drugs (n, %) | 2 (33.3) | 13 (28.3) | 3 (42.9) | 12 (26.7) | 12 (31.6) | 3 (21.4) |
| Age of onset (years) | 64.50 ± 7.34 | 59.70 ± 8.41 | 64.43 ± 7.87 | 59.60 ± 8.35 | 61.18 ± 8.01 | 57.71 ± 9.11 |
| Disease duration (years) | 6.83 ± 4.96 | 4.68 ± 2.94 | 5.29 ± 3.04 | 4.88 ± 3.30 | 5.62 ± 3.32 | 3.07 ± 2.16[##] |
| Hoehn & Yahr stage | 2.58 ± 0.38 | 2.24 ± 0.40 | 2.14 ± 0.38 | 2.30 ± 0.42 | 2.33 ± 0.39 | 2.14 ± 0.46 |
| PIGD-dominant (n, %) | 3 (50.0) | 22 (47.8) | 2 (28.6) | 23 (51.1) | 20 (52.6) | 5 (35.7) |
| MDS-UPDRS I score | 11.83 ± 3.25 | 9.07 ± 4.65 | 11.29 ± 4.92 | 9.09 ± 4.50 | 9.71 ± 4.50 | 8.50 ± 4.83 |
| MDS-UPDRS II score | 13.50 ± 6.09 | 12.13 ± 5.48 | 10.14 ± 6.07 | 12.62 ± 5.41 | 12.79 ± 5.82 | 10.93 ± 4.45 |
| "off-state" MDS-UPDRS III score | 40.17 ± 5.64 | 31.52 ± 11.32[*] | 33.14 ± 8.71 | 32.42 ± 11.54 | 34.63 ± 10.82 | 26.79 ± 10.21[#] |
| best "on-state" MDS-UPDRS III score | 22.00 ± 8.17 | 17.26 ± 7.63 | 17.57 ± 8.70 | 17.84 ± 7.71 | 18.82 ± 8.05 | 15.07 ± 6.38 |
| "off-state" MDS-UPDRS total score | 65.83 ± 11.89 | 53.02 ± 18.11[*] | 54.57 ± 19.07 | 54.49 ± 17.94 | 57.42 ± 18.18 | 46.57 ± 14.96[#] |
| best "on-state" MDS-UPDRS total score | 47.67±13.65 | 38.76 ± 14.68 | 39.00 ± 18.50 | 39.91 ± 14.30 | 41.61 ± 15.51 | 34.86 ± 11.40 |
| Levodopa responsiveness (%) | 45.27 ± 18.92 | 46.07 ± 10.38 | 47.74 ± 18.04 | 45.70 ± 10.29 | 46.85 ± 12.32 | 43.61 ± 8.40 |
| NMS-Quest score | 10.33 ± 4.13 | 7.61 ± 3.76 | 10.29 ± 5.41 | 7.56 ± 3.51 | 8.61 ± 3.81 | 6.07 ± 3.50 |
| SCOPA-AUT cardiovascular domain score | 1.17 ± 1.33 | 0.28 ± 0.89 | 0.86 ± 1.21 | 0.31 ± 0.92 | 0.42 ± 1.06 | 0.29 ± 0.73 |
| SCOPA-AUT gastrointestinal domain score | 7.00 ± 4.34 | 3.65 ± 3.03[*] | 5.43 ± 3.99 | 3.82 ± 3.21 | 4.37 ± 3.32 | 3.14 ± 3.30 |
| SCOPA-AUT total score | 15.67 ± 8.48 | 8.15 ± 6.33[*] | 10.57 ± 7.32 | 8.78 ± 6.95 | 9.34 ± 6.56 | 8.14 ± 8.11 |
| HAMD-17 score | 9.00 ± 4.94 | 5.24 ± 4.28 | 7.43 ± 5.06 | 5.40 ± 4.38 | 5.87 ± 4.19 | 5.14 ± 5.32 |
| HAMA score | 11.83 ± 4.31 | 6.80 ± 4.57[*] | 9.29 ± 4.57 | 7.09 ± 4.80 | 7.50 ± 4.56 | 7.07 ± 5.53 |
| RBD-SQ score | 3.50 ± 3.33 | 1.85 ± 2.82 | 3.43 ± 3.64 | 1.82 ± 2.75 | 2.26 ± 3.09 | 1.43 ± 2.28 |
| PDSS-2 score | 13.00 ± 4.47 | 8.93 ± 6.18 | 12.29 ± 6.52 | 8.96 ± 6.00 | 10.21 ± 5.56 | 7.21 ± 7.20 |
| PDQ-39 score | 23.33 ± 17.61 | 22.70 ± 15.68 | 21.86 ± 15.28 | 22.91 ± 15.96 | 25.05 ± 15.48 | 16.57 ± 15.23 |
| MMSE score | 27.00 ± 4.98 | 27.54 ± 2.96 | 28.86 ± 1.46 | 27.27 ± 3.35 | 27.92 ± 2.59 | 26.29 ± 4.34 |
| MoCA score | 22.67 ± 6.98 | 22.76 ± 4.96 | 23.43 ± 4.47 | 22.64 ± 5.28 | 22.68 ± 4.70 | 22.93 ± 6.40 |
| LEDD (mg) | 504.17 ± 207.62 | 467.37 ± 210.78 | 485.71 ± 131.38 | 469.43 ± 219.34 | 515.44 ± 188.75 | 352.68 ± 220.64[#] |
| Anti-PD drugs | | | | | | |
| Levodopa (n, %) | 6 (100.0) | 44 (95.7) | 7 (100.0) | 43 (95.6) | 38 (100.0) | 12 (85.7) |
| Dopamine agonists (n, %) | 4 (66.7) | 33 (71.7) | 5 (71.4) | 32 (71.1) | 29 (76.3) | 8 (57.1) |
| MAO-B inhibitors (n, %) | 1 (16.7) | 5 (10.9) | 3 (42.9) | 3 (6.7)[$] | 5 (13.2) | 1 (7.1) |

| | | | | | | |
|---|---|---|---|---|---|---|
| COMT inhibitor (n, %) | 0 | 3 (6.5) | 0 | 3 (6.7) | 3 (7.9) | 0 |
| Amantadine (n, %) | 0 | 3 (6.5) | 0 | 3 (6.7) | 2 (5.3) | 1 (7.1) |
| Benzhexol (n, %) | 1 (16.7) | 2 (4.3) | 1 (14.3) | 2 (4.4) | 3 (7.9) | 0 |
| Home BP measurement | | | | | | |
| “off-state” OH (n, %) | 4 (66.7) | 3 (6.5)** | 2 (28.6) | 5 (11.1) | 6 (15.8) | 1 (7.1) |
| “off-state” symptoms (n, %) | 0 | 2 (4.3) | 0 | 2 (4.4) | 2 (5.3) | 0 |
| best “on-state” OH (n, %) | 3 (50.0) | 4 (8.7)* | 1 (14.3) | 6 (13.3) | 6 (15.8) | 1 (7.1) |
| best “on-state” symptoms (n, %) | 2 (33.3) | 6 (13.0) | 1 (14.3) | 7 (15.6) | 8 (21.1) | 0 |
| anti-PD drug-induced hypotension (n, %) | 4 (66.7) | 27 (58.7) | 3 (42.9) | 28 (62.2) | 25 (65.8) | 6 (42.9) |
| LCT BP measurement | | | | | | |
| “off-state” OH (n, %) | - | - | 3 (42.9) | 3 (6.7)$ | 6 (15.8) | 0 |
| “off-state” symptoms (n, %) | 4 (66.7) | 2 (4.3)** | 2 (28.6) | 4 (8.9) | 5 (13.2) | 1 (7.1) |
| best “on-state” OH (n, %) | 3 (50.0) | 4 (8.7)* | - | - | 7 (18.4) | 0 |
| best “on-state” symptoms (n, %) | 4 (66.7) | 6 (13.0)* | 3 (42.9) | 7 (15.6) | 9 (23.7) | 1 (7.1) |
| levodopa-induced hypotension (n, %) | 6 (100.0) | 32 (69.6) | 7 (100.0) | 31 (68.9) | - | - |

Data were shown as mean ± SD or frequency (percentage). Differences between groups were assessed using the Mann–Whitney U test for numerical data and chi-squared test for categorical data.

* Difference in demographic and clinical features between patients with or without OH in the off state, * $P<0.05$, ** $P<0.01$;

$ Difference in demographic and clinical features between patients with or without OH in the best on state, $ $P<0.05$;

# Difference in demographic and clinical features between patients with or without levodopa-induced hypotension in the best on state, # $P<0.05$, ## $P<0.01$.

PD, Parkinson’s disease; “off-state”, defined as the period when all anti-PD drugs were withdrawn for at least 12 hours; OH, orthostatic hypotension; best “on-state”, defined as the peak of anti-PD drugs benefit in the morning at home or the peak of levodopa benefit in the levodopa challenge test at the hospital; levodopa-induced hypotension, was defined, if either one of following criteria was met: (1) a decrease of systolic BP by at least 20 mmHg/diastolic BP by 10 mmHg from “off-state” to the best “on-state” in the supine position; (2) a decrease of systolic BP by at least 20 mmHg/diastolic BP by 10 mmHg from “off-state” to the best “on-state” in the 1-min/3-min standing position; BMI, Body Mass Index; PIGD, postural

instability/gait difficulty; MDS-UPDRS, Movement Disorder Society-Unified Parkinson's Disease Rating Scale, "off-state" and best "on-state" MDS-UPDRS III and total score were evaluated in levodopa challenge test (LCT); levodopa responsiveness (%), defined as $Levodopa\ Responsiveness\ (\%) = \frac{off\ state\ MDS\ UPDRS\ III\ scores - best\ on\ state\ MDS\ UPDRS\ III\ scores}{off\ state\ MDS\ UPDRS\ III\ scores} \times 100\%$; NMS-Quest, Non-Motor Symptoms Quest Scale; SCOPA-AUT, Scale for Outcomes in Parkinson's Disease-Autonomic; HAMD-17, Hamilton Depression Scale; HAMA, Hamilton Anxiety Rating Scale; RBD-SQ, Rapid Eye Movement (REM) Sleep Behavior Disorder Screening Questionnaire; PDSS-2, Parkinson's Disease Sleep Scale-2; PDQ-39, 39-item Parkinson's Disease Questionnaire; MMSE, Mini Mental State Examination; MoCA, Montreal Cognitive Assessment; LEDD, levodopa equivalent daily dosage; MAO-B, monoamine oxidase B; COMT, catechol-O-methyltransferase; BP, blood pressure; symptoms, defined as dizziness, sleepiness, or blurred vision experienced by PD patients within 3 minutes in upright posture.
